# Tobacco Amblyopia and Tachycardia

**Published:** 1905-04-15

**Authors:** 


### TOBACCO AMBLYOPIA AND TACHYCARDIA.

Mr. Chas. Wray[1] draws attention to the frequent association of amblyopia and tachycardia as a result of the use of tobacco. Either symptom may exist alone, but in ophthalmic practice it is frequent to find that they are combined. The amblyopia is probably due to saturation of the blood with tobacco alkaloids; its occurrence in some cases and not in others may be due less to idiosyncrasy than to the state of the teeth, which in many smokers are kept in a bad condition. The tachycardia is doubtless a result of the action of the poison in some part of the nervous mechanism of the heart. A practical question is whether the disuse of tobacco will cause the disappearance of the amblyopia and the tachycardia. In early cases abstinence is usually followed by return of vision, but the convalescence is a protracted one, and generally extends over many weeks. The outlook in regard to tachycardia is even less satisfactory, for this symptom, when once established, may continue to be troublesome for many years even though the use of tobacco is completely abandoned. In cases of amblyopia the medicinal treatment usually adopted is potassium iodide and strychnine, but the value of these remedies Mr. Wray ventures to doubt. He proposes, on the basis of the fact that nicotine is freely soluble in water, to use large draughts of water in order to secure the elimination of the poison. He, therefore, orders sufferers from tobacco amblyopia to dress warmly and to drink a pint of hot water at 7 a.m., this to be followed by a brisk walk in the open air; the draught and the exercise are repeated a second time, and then after a few minutes' rest breakfast is taken. In the middle of the forenoon and also in the afternoon a pint of warm water is again prescribed. As a result of this treatment some patients regained practically normal vision in the course of fourteen days, whereas, as is well known, the more usual medicinal method hardly expects this result under a period of two months or more. Moreover, some of the patients in this series continued to use tobacco to the extent even of a quarter of an ounce per diem. The effect of this water treatment on tachycardia is more doubtful; there seems reason to think that when this symptom has appeared as a result of tobacco some change has been produced in the nervous system which is almost, if not quite, beyond the limit of recovery. Altogether Mr. Wray's communication is an interesting and instructive one, and his claim that ophthalmic surgeons who undertake the treatment of tobacco amblyopia and other medical conditions should look out for other symptoms than those due to defects of the visual apparatus rests on a sound basis.

[1] Lancet, Feb. 18, 1905.

